# Evidence for antimicrobial stewardship and reduced antimicrobial resistance in the Mid-West of Ireland, 2012 to 2023: findings from a One Health study

**DOI:** 10.2807/1560-7917.ES.2025.30.13.2400512

**Published:** 2025-04-03

**Authors:** James Powell, Santosh Sharma, Alan Johnson, Siobhan Barrett, Caroline Garvan, Nuala H O’Connell, Colum P Dunne

**Affiliations:** 1 Department of Microbiology, University Hospital Limerick, Limerick, Ireland; 2 School of Medicine and Centre for Interventions in Infection, Inflammation, and Immunity (4i), University of Limerick, Limerick, Ireland; 3 Regional Veterinary Laboratory, Knockalisheen, Limerick, Ireland; 4 Pharmacy Department, University Hospital Limerick, Limerick, Ireland; 5 Department of Agriculture, Food and the Marine, Backweston Campus, Celbridge, Kildare, Ireland; 6 School of Pharmacy, Queen’s University Belfast, Belfast, Northern Ireland

**Keywords:** E. coli, Escherichia, AMR, antimicrobial, resistance, Ireland, bovine, One Health

## Abstract

**Background:**

*Escherichia coli*, a pathogen commonly infecting humans and bovines, is a prime sentinel indicator and predictor for antimicrobial resistance (AMR). Tracking epidemiological trends of AMR is essential to address this global One Health threat.

**Aim:**

To perform a comprehensive retrospective epidemiological analysis of AMR trends in *E. coli* isolated from human urine and blood and bovine specimens, and compare with antimicrobial consumption or sales data for humans.

**Methods:**

All *E. coli* isolates with susceptibility results from human urine (n = 122,419), blood (n = 2,373) and bovine specimens (n = 585) from 2012–23 in the Mid-West of Ireland were analysed. The resistance trends of nine commonly used antimicrobials were compared with their consumption by humans or sales in community and hospital settings.

**Results:**

Over the 12-year period, resistance against common antimicrobials was lowest among the bovine isolates (range: 2–44%). Human urine isolates showed lower resistance (5–59%) than bloodstream isolates (12–69%). There was a downward trend in resistance to all antimicrobials between 2012 and 2023 in the human isolates (p < 0.001), except for piperacillin/tazobactam where resistance increased, in each case correlating with antimicrobial usage. Bovine isolates demonstrated reduced resistance to co-amoxiclav (p = 0.001), with no trend observed for other antimicrobials.

**Conclusion:**

Our data showed reduced resistance to many antimicrobials for *E. coli* from human and bovine populations in our region. Increased use of ‘preferred’ antimicrobials in humans and reduced use of those ‘to be avoided’ was observed. The findings indicate the emerging effectiveness of AMR strategies and highlight the value of One Health AMR.

**Key public health message**

**What did you want to address in this study and why?**

Antimicrobial resistance (AMR) is a major global challenge, driven by the overuse of antimicrobials in human and animal medicine. Using a One Health approach (i.e. linking human, animal and environmental data), we examined AMR trends in *Escherichia coli* detected from human and bovine clinical specimens in the Mid-West of Ireland from 2012 to 2023 and compared these trends to the overall use of antimicrobials in humans.

**What** **have we learnt from this study?**

Over the 12-year study period, we observed a statistically significant downward trend in antimicrobial resistance (1% to 24% decrease) in human samples except for piperacillin/tazobactam (3% increase). Consumption of antimicrobials ‘to be avoided’ decreased, and of ‘preferred’ antimicrobials increased. In the bovine isolates, resistance was lower than in human samples, but overall downward trends were not statistically significant.

**What are the implications of your findings for public health?**

This study provides important evidence that antimicrobial stewardship and other strategies are having a measurable effect in addressing AMR. The findings highlight the necessity of ongoing surveillance and a One Health approach to address this global health threat.

## Introduction

*Escherichia coli* is one of the most common bacterial pathogens in humans [1] and is predominantly associated with urinary tract and bloodstream infections [2]. It is also a leading cause of clinical mastitis and metritis in dairy bovines, often linked to severe infections [3,4]. Notably, *E. coli* is considered to be an excellent sentinel and predictor of antimicrobial resistance (AMR) for these species [5] as well as many other species and the environment, making it a suitable target pathogen for One Health AMR surveillance [6]. It is well established that antimicrobial use in humans is linked to increased AMR [7]. The agricultural and food sectors are also implicated [8], and AMR can be transferred from animals to humans by direct contact or via the food chain and the environment [9].

Global antibiotic consumption by humans increased by 39% from 2000 to 2015, most of the growth occurring in low- and middle-income countries (LMIC), correlated with increased gross domestic product per capita [10]. There is a demonstrated correlation between antimicrobial consumption and the emergence of resistance [11]. Conversely, stabilising or decreasing trends in the consumption of some antimicrobials have been associated with concurrent stabilisation in resistance rates to those antimicrobials [12]. Therefore, the primary strategy employed to address AMR involves reducing antimicrobial use in both human healthcare and livestock production [13].

Global antibiotic consumption in animals, on the contrary, has reduced considerably in recent years, particularly those critical to human medicine, e.g. quinolones, third- and fourth-generation cephalosporins, macrolides and polymyxins. The use of antimicrobials for growth promotion has been phased out in most regions, in conjunction with increased awareness, improved veterinary practices, regulatory measures and international collaboration [14]. Global sales of veterinary antibiotics have also been targeted for reduction: in Europe, the European Commission ‘Farm to Fork Strategy’ has a commitment to reduce European Union/European Economic Area (EU/EEA) sales of antimicrobials for farmed animals and aquaculture by 50% from 2018 to 2030 [15]. In the 4 years from 2018 to 2022, there has already been a 30% reduction for the EU/EEA and a 23% reduction for Ireland [16], with particular focus on oral medication in the intensive agricultural sectors and intramammary dry cow antibiotics in tandem with disease prevention measures and the optimisation of vaccine use [17].

Ireland has actively contributed to the European Commission’s plan to reduce AMR with the launch of its first One Health national action plan to address antimicrobial resistance (iNAP) in October 2017 [18], and its second action plan (iNAP2) in October 2021 [19]. Surveillance is one of the key interventions recommended for the control of AMR [20]. The European Commission produces regular inter-agency reports with extensive data on AMR and antimicrobial usage [21,22]. However, independent academic studies comparing the dynamics of AMR in humans and animals are rare [5]. In addition, there is a paucity of epidemiological AMR studies in Ireland. Most, including studies from drinking water [23], groundwater [24], bathing water [25,26], pigs [27], poultry [28] and farm waste [29] consist of point-prevalence studies so do not demonstrate temporal trends. Three recent epidemiological AMR studies of *E. coli* from human urine in Ireland are available [30-32], one showing increasing resistance, the other two showing stabilisation.

The aim of our study was to provide an epidemiological analysis of AMR in the Mid-West of Ireland over a 12-year period on human urine and blood specimens from both community and acute hospital settings, and bovine specimens from a regional veterinary laboratory. This selection constitutes the most common specimen types in which *E. coli* is detected and susceptibility testing is performed, offering a comprehensive overview of AMR across healthcare, community and animal sectors, and supporting a One Health approach to AMR surveillance. 

## Methods

### Study setting for laboratory testing

The Department of Clinical Microbiology at University Hospital Limerick (UHL), Health Services Executive (HSE) Mid-West, provides a centralised microbiology service for six acute hospital sites in the Mid-West of Ireland, an administrative region encompassing the counties Limerick, Clare and North Tipperary. The hospital group comprises one tertiary care teaching hospital, one maternity hospital, one specialist orthopaedic hospital and three general hospitals (currently 871 total beds). These six acute hospitals, nine community healthcare facilities, in addition to more than 60 nursing homes and approximately 150 general practice surgeries, cater for a population of 473,269 people [33]. All human data were stored on a single laboratory information management system (LIMS) at UHL.

The Department of Agriculture, Food and the Marine (DAFM) in Ireland, through its Regional Veterinary Laboratory (RVL) network carries out continuous surveillance for exotic and endemic diseases in Irish livestock. To facilitate this, it provides a laboratory diagnostic service to private veterinary practitioners and farmers. The RVL in Limerick provides the service for the Mid-West of Ireland, a region with just over 1,000,000 cattle, which is ca 17 per cent of the cattle population of the Republic of Ireland. 

For this study, all *E. coli* susceptibility results from 2012 to 2023 were extracted from the LIMS of HSE-Mid-West and that of the RVL. The antimicrobials analysed were those routinely tested by the laboratories, identified as the most appropriate for treating infections associated with these specimen types.

### Clinical diagnostic specimens 

#### Human specimens

The specimen types selected for these *E. coli* isolates were human urine and blood samples requested from all locations. ‘Community’ urine isolates were classified based on the specimen requesting clinician type, i.e. general practitioners, which also included nursing homes. ‘Hospital’ urine isolates were classified based on the patient location, i.e. acute hospital in-patient locations. All bloodstream isolates were from acute hospital in-patient locations. Isolates were classified as ‘hospital-acquired’ blood isolates if the specimen was collected more than 48 h post-admission. ‘Community-acquired’ blood isolates were detected from specimens collected within 48 h of admission. 

All urine isolates were included in the study, regardless of colony count, leucocyte count or duplication of specimen testing. No clinical information was available on the patients. However, in our laboratory, susceptibility testing is only performed if the colony count and/or leucocyte count are indicative of likely infection. 

Thirty-day mortality data were extracted from the hospital's patient management system, which records both in-hospital deaths and the majority of external deaths; it may lack data on a small number of external cases, however. 

See Supplementary Tables S1 and S2 for information on the demographics of patients from whom the urine and blood samples were obtained. 

#### Bovine specimens

All *E. coli* isolates from bovine specimens i.e. milk (68% of specimens, 399/585), liver, lung and other internal organs (20%, 117/585), faeces (10%, 60/585) and other (2%, 9/585) specimens submitted for analysis at the RVL were included. These specimens were either submitted by veterinary surgeons from unwell animals (mastitis being the most common presentation) or collected post-mortem from deceased animals. 

### Antimicrobial selection and susceptibility testing

The antimicrobials analysed were those routinely tested by the laboratories, identified as the most appropriate for treating Gram-negative infections associated with these specimen types. See the Table for the antimicrobials tested for each specimen type. 

**Table:** Gram-negative antimicrobials tested per specimen type, comparable across each antimicrobial class, Mid-West of Ireland, 2012–2023

Antimicrobial class	Human urine	Human blood	Bovine
Aminopenicillin	Amoxicillin	Amoxicillin	Ampicillin
Co-trimoxazole	N/A	Co-trimoxazole	Co-trimoxazole
Beta-lactam and inhibitor	Co-amoxiclav	Co-amoxiclav	Co-amoxiclav
	Piperacillin/tazobactam	Piperacillin/tazobactam	N/A
Quinolone	Ciprofloxacin	Ciprofloxacin	Enrofloxacin
1st-gen. cephalosporin	Cephalexin	N/A	Cephalothin
3rd-gen. cephalosporin	Cefpodoxime	Ceftriaxone	Ceftiofur
Aminoglycoside	Gentamicin	Gentamicin	N/A
Aztreonam	Aztreonam	Aztreonam	N/A

gen.: generation; N/A: not applicable, no test result available for this category of specimen.

Closely related antimicrobials were compared across the specimen types. Co-amoxiclav (amoxicillin, an aminopenicillin with the beta lactam inhibitor clavulanic acid) susceptibility was directly comparable across all categories of human and bovine isolates. Aminopenicillins, quinolones, first-generation cephalosporins, and third-generation cephalosporins were considered equivalent for category comparisons; these antimicrobial classes are used to describe the results, instead of the individual agents. Gentamicin, piperacillin/tazobactam and aztreonam were not tested for the bovine isolates, so for these antimicrobials, comparisons could only be made between the different categories of human specimens. Co-trimoxazole was not tested for human urine isolates, and first-generation cephalosporins were not tested for human bloodstream isolates.

Supplementary Tables S3, S4, S5, S6, S7 and S8 for full details including aggregate susceptibilities per antimicrobial for each individual year and for hospital- and community-sourced human specimens. 

Susceptibility testing of human isolates was performed on the Sensititre instrument (Trek Diagnostic systems, ThermoFisher Scientific) using EUCAST criteria [34]. There were a small number of changes (each no more than one dilution difference) made to the EUCAST breakpoint criteria during the study period, as outlined in Supplementary Figure S1. Susceptibility testing of bovine isolates was performed using disk diffusion and interpreted according to EUCAST guidelines where available. The susceptibility results for the antimicrobials tested for each year of the study period are listed in Supplementary Table S9. The antimicrobials which were comparable with those tested on human isolates are listed in the Table.

### Antimicrobial consumption data

Human hospital antimicrobial consumption rates from 2007 to 2022 were generated from the Health Protection Surveillance Centre MicroB Project [35]. These data were available for each individual hospital; the total for the group of hospitals (referred to as `local data’) was calculated by multiplying each of the six acute hospital's defined daily doses (DDDs) by its bed days (BDU), summing these products, and then dividing by the total BDU for the group. 

National data used in this study consisted of reported rates for the total acute hospitals of Ireland. National community antimicrobial consumption data were supplied by the Primary Care Reimbursement Service (PCRS), and separate data for four reimbursement schemes were pooled for this study: the Drug Payment Scheme, the General Medical Services Scheme, the Long-Term Illness Scheme, and also data for High-Tech Drugs (in total, covering ca 40% of the population in 2023). The DDDs of these antimicrobials were calculated by multiplying the quantity of active ingredient in grams by the quantity sold and dividing by the latest DDD figures [36]. 

While information on veterinary antimicrobials generally is available for Ireland [17], species level antimicrobial consumption data were not available for bovines.

### Data analysis

Data were collated using Microsoft Excel. Descriptive statistics, including frequency with percentages, were used to estimate trends in antibiotic resistance over 12 years. The Cochran-Armitage test for trend was applied to determine whether there were increasing or decreasing trends in resistance over this period. The degree and significance of these resistance trends were calculated for each antibiotic. We considered a p value < 0.05 as statistically significant. Analyses were performed using MS Excel and STATA 17.0 (Stata Corp.).

## Results

During the 12-year period, susceptibility tests were performed on 122,419 human urine isolates, 2,373 human bloodstream isolates and 585 bovine isolates. The urine isolates were predominantly from individuals aged ≥ 15 years (90%, n = 110,296; mean age: 58 years; range: < 10 days–105 years), females (85%, n = 104,586; males 15%, n = 17,781; unknown < 1%, n = 52) and from specimens requested by general practice (67%, n = 82,516; acute hospital patients: 29%, n = 36,038 and other: 3%, n = 3,865). The bloodstream isolates from acute hospital patients were almost exclusively from individuals aged ≥ 15 years (98%, n = 2,319; mean age: 70 years (range: < 10 days–102 years), and had an almost equal sex distribution (52% female, n = 1,228 and 48% male, n = 1,145). The proportion deemed to be ‘hospital-acquired’ was 19% (n = 450). The 30-day mortality for patients with urine isolates was 1% (133/11,295) and bloodstream isolates was 9% (20/234, crude mortality from all causes, 2023 data).

In 2023, in the overall clinical context, *E. coli* accounted for 61% and 25% of all urine and blood isolates detected in our laboratory, respectively. Supplementary Figures S2 and S3 provide an overview of all pathogens isolated from urine and blood specimens. In the same year, *E. coli* represented 31% of bovine isolates identified; the species detected in bovine isolates are provided in Supplementary Figure S4. 

### Comparison of antimicrobial resistance between specimen types

**Figure 1:**
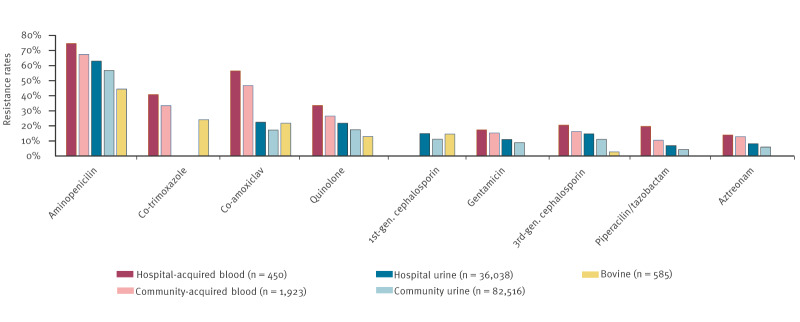
Comparison of overall resistance rates for *Escherichia coli* isolates from human and bovine clinical specimens from the Mid-West of Ireland, 2012–2023

For the 2012–23 study period, the rates of antimicrobial resistance in the bloodstream isolates were higher than that of the urine isolates, across all seven comparable antimicrobials. Figure 1 provides an overview of the resistance rates to various antimicrobials for *E. coli* isolates sourced from (i) hospital-acquired and (ii) community-acquired blood specimens, (iii) urine specimens requested from acute hospital locations, (iv) urine specimens requested by general practitioners in the community and (v) bovine clinical diagnostic specimens. The resistance rates of the human urine isolates were higher than the bovine isolates for the following antimicrobials: aminopenicillin (p < 0.001), quinolone (p < 0.001, ciprofloxacin vs enrofloxacin) and cefpodoxime (third-generation cephalosporin p < 0.001). The bovine isolates had higher rates of resistance to co-amoxiclav (not statistically significant) and first-generation cephalosporins (cephalothin vs cephalexin, not statistically significant) than the urine isolates. Predictably, within the bloodstream isolates, resistance rates were higher among the hospital-acquired isolates compared with the community-acquired isolates for all eight antimicrobials examined (p < 0.001). Similarly, the resistance rates from the acute hospital urine isolates were higher than for the community isolates across the full panel of antimicrobials (p < 0.001). 

gen.: generation.

Quinolone antimicrobials were ciprofloxacin for human isolates, enrofloxacin for bovine isolates. First-generation cephalosporin antimicrobials were cephalexin for human urine (not tested for human blood) isolates, and cephalothin for bovine isolates. Third-generation cephalosporin antimicrobials were ceftriaxone for human blood isolates, cefpodoxime for human urine and for bovine isolates. Co-trimoxazole was not tested for urine isolates.

### Temporal resistance trends in the study period

**Figure 2:**
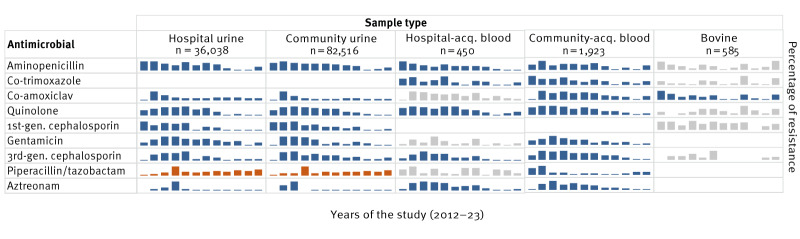
Sparkline graphs depicting antimicrobial resistance for *Escherichia coli* in human urine and blood specimens, and bovine specimens in the Mid-West of Ireland, 2012–2023

During the 12-year study period, a decrease in resistance was noted across all types of specimens examined, with nearly all antimicrobials showing this trend (Figure 2, see also Supplementary Tables S3–9 for trends for each antimicrobial in each specimen category).

acq.: acquired; gen.: generation.

These sparkline graphs depict the percentage of resistance for each of the specimen types: human urine (n = 118,554) human blood (n = 2,373), and bovine specimens (n = 585). The 12 columns on the X-axis represent each of the 12 years of the study (2012–23), and the Y-axis represents the percentage of resistance to each antimicrobial. Charts with bars in dark blue show statistically significant reductions in resistance rates, charts with bars in red show statistically significant increases, and charts with bars in grey are not statistically significant.

Aminopenicillin antimicrobials were amoxicillin for human isolates, ampicillin for bovine isolates. First-generation cephalosporin antimicrobials were cephalexin for human urine (not tested for human blood) isolates, and cephalothin for bovine isolates. Third-generation cephalosporin antimicrobials were ceftriaxone for human blood isolates, cefpodoxime for human urine and for bovine isolates. Co-trimoxazole was not tested for urine isolates. Quinolone antimicrobials were ciprofloxacin for human isolates, enrofloxacin for bovine isolates.

Among the eight antimicrobials analysed for the urine isolates, seven exhibited a statistically significant reduction of resistance over the 12-year study: aminopenicillin, co-amoxiclav, ciprofloxacin, cefpodoxime, cephalexin, gentamicin and aztreonam. Piperacillin/tazobactam showed increased resistance. No difference in the temporal trend of antimicrobial resistance was found between urine isolates from community patients and those from acute hospital settings.

A similar trend was evident for the blood isolates, and a statistically significant reduction in resistance was observed for all antimicrobials when examining all blood isolates, i.e. hospital-acquired and community-acquired isolates combined. This pattern was also evident within the hospital-acquired and community-acquired isolates separately, although statistical significance was not achieved for certain antimicrobial categories within the hospital-acquired isolates.

In the bovine isolates, co-amoxiclav showed a statistically significant reduction in resistance over the study period (Figure 2). None of the other antimicrobials demonstrated a statistically significant trend in either an upward or downward direction; however, this lack of significance may be attributable to the limited sample size. 

### Antimicrobial consumption

Local and national human acute hospital antimicrobial consumption figures from 2007 to 2022 are available in Supplementary Table S10. The local trends were aligned with national patterns for most antimicrobials. Both local and national data indicated a decreased use of quinolones, but increased use of third-generation cephalosporins, carbapenems, co-amoxiclav and piperacillin/tazobactam. Gentamicin usage decreased nationally but showed an increase in our hospitals.

**Figure 3:**
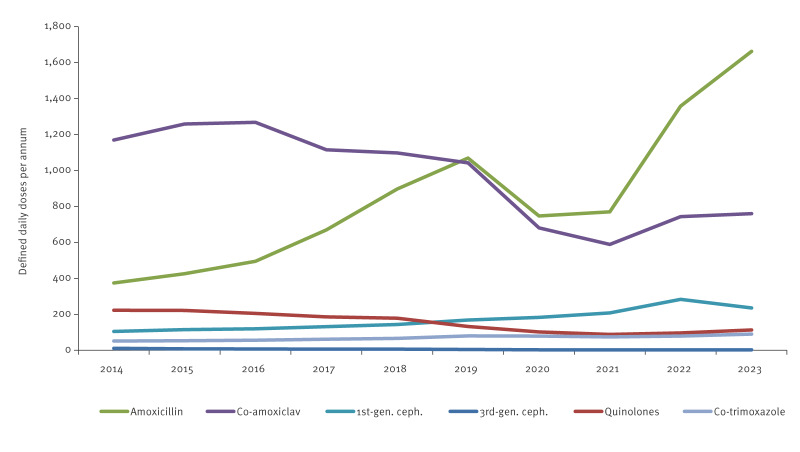
Defined daily doses per annum of selected Gram-negative antimicrobials dispensed for human use in community healthcare in Ireland, 2014–2023, (n = ~ 2.1 million people)^a^

National data of community pharmaceuticals (PCRS data, Supplementary Figure S5) from 2014 to 2023 show that amoxicillin and co-amoxiclav were consistently the most commonly dispensed antimicrobials, representing roughly half of all antimicrobials (in kg of active ingredient). The ratio between these two antimicrobials shifted dramatically, however, from more than three times as much co-amoxiclav as amoxicillin in 2014 (8.1 vs 2.6 tons; 3.6 vs 1.1 million DDDs) to less than half as much co-amoxiclav as amoxicillin in 2023 (5.7 vs 12.4 tons; 2.5 vs 5.5 million DDDs). Supplementary Figure S5 provides a comparison of antimicrobials dispensed in 2014–23. In this instance, however, while the change in ratio between co-amoxiclav and amoxicillin occurred to some degree because of a 30% reduction in the former (8.1 to 5.7 tons; 3.6 million to 2.5 million DDDs), a larger factor was the more than fourfold increase in the use of the latter (2.6 to 12.4 tons; 1.1 to 5.5 million DDDs). This trend can be seen clearly in Figure 3, which also shows that consumption of third-generation cephalosporins and quinolones exhibit a downward trend, and first-generation cephalosporins and co-trimoxazole show an increasing trend.

ceph.: cephalosporin; gen.: generation.

^a^ The population figure of 2.1 million people represents 40% of the median population of Ireland from 2012 to 2023.

National community antimicrobial consumption data were supplied by the Primary Care Reimbursement Service (PCRS), with separate data for four reimbursement schemes which were pooled for this study: The Drug Payment Scheme, the General Medical Services Scheme, the Long-Term Illness Scheme also data for High-Tech Drugs (in total, covering ca 40% of the population in 2023).

## Discussion

The penicillin (including aminopenicillins) class of antimicrobials is the most commonly used class for human infections [37] and the second most common veterinary [38] antimicrobial (after tetracyclines) in the EU/EEA. In terms of human use, Ireland ranks fourth in community consumption and seventh in hospital consumption of penicillin antimicrobials in the EU/EEA, when adjusted for population size [37]. Consequently, it is unsurprising that our study showed the highest rate of resistance to aminopenicillin, with 59% of bloodstream isolates from 2022 (63% of hospital-acquired, 58% of community-acquired strains) exhibiting resistance. This compares with a national bloodstream resistance rate of 62% and an EU/EEA median rate of 53.5% (range: 32.4–68.6) for the same year [39]. Resistance in urine isolates was 56% in 2022 (60% of hospital isolates, 54% of community isolates) in our study. Another Irish study of community urine isolates had a resistance rate of 48.5% in that year [30]. Similarly, the resistance figures for hospital and community urine isolates in our study in 2014 were 64% and 58% (see Supplementary Tables S7 and S8), just below those from a Dublin study of that year (71% and 60%, respectively [32]). For comparison, rates from similar studies of urine isolates include France (42.5%, unspecified) [5], Australia (52% hospital and 42% community) [40] and the United Kingdom (UK) (57% hospital and 48% community) [41]. 

Aminopenicillin resistance in the bovine isolates in our study was 44%, the highest resistance rate of all antimicrobials tested for the bovine isolates. From 78 studies in resistancebank.org [42] with bovine *E. coli* aminopenicillin prevalence rates, the median prevalence rates across the EU/EEA, Africa, Asia and the Americas were 49.5%, 80%, 67% and 54%, respectively.

The presence of a beta-lactamase inhibitor in combination with aminopenicillin significantly decreased the rate of resistance detected, most notably in the urine isolates. In 2022, 37% of bloodstream isolates (46% of hospital-acquired, 35% of community-acquired strains) were resistant to co-amoxiclav, compared with just 16.5% of urine isolates (19% of hospital isolates, 14% of community isolates, see Supplementary Tables S3–S8). The Irish bloodstream *E. coli* co-amoxiclav resistance rate [43] in 2022 was 45%; no EU/EEA bloodstream resistance data [39] are available and there are limited data available in the literature. Published resistance rates from urine isolates range from 17% to 48% from Irish studies [30-32] to as low as 4% in Australia [40] and the UK [41], and over 80% in India [44]. Resistance to co-amoxiclav in the bovine isolates was 7% in 2022, compared with 42% in 2012. Median prevalence rates from bovine studies internationally range from 49% in South and Central America to 83% in Asia and 89% in Africa; there are no data for the EU/EEA or the United States [42].

The antimicrobial with the greatest decrease of resistance for human isolates in the 12-year study period was ciprofloxacin; there was a decrease of 24% (p < 0.001) in the bloodstream isolates and 10% in the urine isolates (p < 0.001). Conversely, quinolone resistance for bovine isolates showed an increase of 9%, but this increase was not statistically significant (p = 0.052). The prevalence of resistance of our bloodstream isolates in 2022 (16% total, 21% hospital-acquired, 15% community-acquired) was identical to that seen in the national figure for that year (16%) [43], and slightly lower than the median European figure of 18% [39]. Two European countries had resistance rates over 40% [39], and similarly high rates are also found in many African countries [45]. In 2022, quinolone resistance among urine isolates was 14% (16.5% for hospital isolates, 13% for community isolates). For bovine isolates, it was 4% in 2022 but rose to 21% in 2023.

Data on bloodstream isolates for first-generation cephalosporins were unavailable as these agents are not routinely tested for such infections in our institution. Cephalexin resistance among urine isolates was 11% in 2022 (13% in hospital isolates, 10% in community isolates), and cephalothin resistance among bovine isolates that year was 9%. Over the study period, ceftriaxone exhibited the second most substantial reduction in resistance among blood isolates, decreasing by 17% (p < 0.001), and maintaining resistance rates below 10% in the last two years. In 2022, the resistance rate was 8%, with community isolates exhibiting higher resistance (not statistically significant) compared with hospital isolates (6.1% in hospitals, 8.6% in communities). However, this trend was not consistent throughout the entire study period, where resistance rates from 2012 to 2013 were higher in hospital isolates (21%) compared with community isolates (16%). Third-generation cephalosporin resistance rates were lower in the urine isolates for cefpodoxime (2022 data): 11% resistance (13% in hospital isolates, 10% in community isolates) with a 4% decrease over the study period (p < 0.001). The national bloodstream resistance rate for 2022 was 10%, just below the median European rate (12%). Cefpodoxime resistance in the bovine isolates was 2% in 2022, which followed after 4 consecutive years with no resistance detected. The use of third-generation cephalosporins in veterinary medicine is rare, accounting for less than 1% of antimicrobial usage in food-producing animals in Ireland and in the EU/EEA (0.3% and 0.2% respectively, 2022 data) [38]; this may explain the low resistance seen in that population.

Gentamicin resistance has also been in decline in the urine and blood isolates of our study (13% and 3% reductions, respectively). The 2022 bloodstream isolate resistance rate in our study was 8% (9% for hospital isolates, 7% for community isolates), similar to the national rate (8.6%) and the median European rate (8.6%). Gentamicin resistance in urine isolates in our study in 2022 was also 8% for hospital and community isolates. Aminoglycoside resistance was analysed for the bovine isolates, and in 2022 the resistance to kanamycin and neomycin was 9% and 11% respectively. Both antimicrobials showed decreasing trends in resistance, which was statistically significant for neomycin (p < 0.005). Aminoglycosides were the fourth most commonly used veterinary antimicrobial in 2022, consisting of 7% of total sales for food producing animals [17].

Resistance to co-trimoxazole showed a 15% decline (p < 0.001) in the bloodstream isolates, 3 of the first 4 years had resistance above 40%, 2 of the last 4 years were below 30%. The 2022 bloodstream resistance rate was 28% (30% hospital isolates, 27% community isolates), no national or European antimicrobial resistance or antimicrobial consumption data are available.

Piperacillin/tazobactam is the most commonly used intravenous antimicrobial in our hospital, consumption has more than doubled over the study period and was 45% greater than the median national hospital usage rate in 2022. Resistance to this antimicrobial among urine isolates has also increased, among both isolates from the hospital (6% increase, p < 0.001) and community (4% increase, p < 0.001). Paradoxically, resistance has not risen for bloodstream isolates, in fact there was a decline of 9% (p < 0.001) over the study period. The reduction in resistance was statistically significant only for community isolates, however (10% decline, p < 0.001). There was a decline of resistance in hospital-acquired isolates, (3%) but it was not statistically significant (p > 0.5). The 2022 resistance rate was 13% (21% hospital isolates, 11% community isolates), similar to national resistance (11.6%; no European data). This antimicrobial was not tested for bovine isolates.

The resistance rates of bloodstream isolates in our region are shown to be very similar to national rates, which are in turn very close to EU/EEA median rates. Some disparities were identified however: Aminopenicillin resistance in Ireland was shown to be higher than the EU/EEA median for bloodstream isolates, and the rates for urine and bovine isolates was higher than many international publications. In this study, we showed a dramatic increase of aminopenicillin prescribing in the community from 374 DDDs per inhabitant in 2014 to 1,662 in 2023, with the largest increase seen in the post-COVID-19 pandemic period. Despite this increased community consumption, resistance rates declined in all of the specimen groups examined (statistically significant in human isolates, not statistically significant in bovine isolates).

Our study also highlighted a reduction in co-amoxiclav resistance which was statistically significant among the bovine isolates and all of the human specimens apart from hospital-acquired bloodstream isolates. The reduction of resistance among community human isolates correlates with our data which demonstrated a reduction in co-amoxiclav prescriptions in that group, in line with government recommendations advocating for a transition from ‘antibiotics to be avoided’ (such as co-amoxiclav) to ‘preferred antibiotics’ (such as amoxicillin), as outlined in urinary tract infection (UTI) treatment guidelines [46], the Green Red Antibiotic Quality Improvement Initiative For Community Prescribers [47] and the iNAP2 policy document [19]. This may serve as evidence indicating the successful implementation of an antimicrobial stewardship measure.

Further evidence of effective antimicrobial stewardship in the community setting is reflected in the decreased consumption of second- and third-generation cephalosporins and quinolones (‘antibiotics to be avoided’), which was matched by decreased resistance. However, there was also reduced resistance to first-generation cephalosporins (‘preferred antibiotic’) in our community and acute hospital isolates, despite a slight increase in consumption. In the EU/EEA, targeted efforts have also successfully reduced the veterinary use of third- and fourth-generation cephalosporins and quinolones: from 2011 to 2022, there has been a 39% and 44% reduction respectively, and in 2022 less than 1% of veterinary antimicrobials used in Ireland were in these categories [48]. Nevertheless, our study found no statistically significant change in resistance to these antimicrobials among the bovine isolates, which may be attributed to the limited sample size.

Many ecological studies have shown strong correlation between antimicrobial consumption and the development of resistance in ambulatory, primary care and acute hospital settings [49]; This effect was evident in our study by the increased usage and resistance of piperacillin/tazobactam. Evidence of the reverse effect is rare; most countries are in an upward or stable trajectory for antimicrobial resistance, both LMICs [50], and also many high-income countries (HICs) such as the United States [51]. Antimicrobial consumption remains very high in LMICs, with fewer reports of antimicrobial stewardship programmes (ASPs) from these countries. In a study of 52 ASPs worldwide, only four were from LMICs: three from China and one from Iran [52].

Limitations of our study include evolving breakpoint criteria over the study period, with no MIC or zone size data available to allow better comparison of the data. Nevertheless, a singular breakpoint alteration would not impact the statistical analysis. The small sample size of bovine isolates resulted in low statistical power. Susceptibility results were not de-duplicated per patient, which may lead to overestimation of resistance rates from repeat submission of specimens from recalcitrant infections. Antimicrobial consumption or sales data were not available for bovines as there are no species-specific veterinary data collected in Ireland. The study did not account for patient age, which may affect the generalisability of the findings. The study period included the COVID-19 pandemic, which may have influenced antimicrobial resistance patterns and healthcare practices.

## Conclusion

This unique cross-sector One Health study of antimicrobial resistance in *E. coli* from human and bovine clinical isolates showed decreasing resistance across a number of different antimicrobial classes. The resistance pattern across all five sample types—lowest in bovine isolates, then community urine, hospital urine, community-acquired bloodstream, and highest in hospital-acquired bloodstream—illustrates the effect of varying antibiotic pressures in these sectors. A decrease in AMR brings considerable benefits to public health by reducing the prevalence of drug-resistant infections; it enhances healthcare sustainability by preserving the effectiveness of antibiotics, and economically, it contributes to productivity gains and avoids the expenses associated with managing resistant infections. Therefore, the findings of this study validate ongoing national and international efforts to combat AMR, as they demonstrate measurable progress in the fight against antibiotic resistance.

## Supplementary Data

1232000 Supplement
