# Supplementary material for: Evidence for antimicrobial stewardship and reduced antimicrobial resistance in the Mid-West of Ireland, 2012 to 2023: findings from a One Health study
Source: Euro Surveill. 2025 Apr 3;30(13):2400512. doi: 10.2807/1560-7917.ES.2025.30.13.2400512 (PMC11969961; doi:10.2807/1560-7917.ES.2025.30.13.2400512)
Supplement: Supplement [file 24-00512_DUNNE_Supplement.pdf]

Supplementary information for the following publication: Evidence for Antimicrobial Stewardship and Reduced Antimicrobial Resistance: Findings from a One Health Study in Ireland

Disclaimer:

This supplementary material is hosted by *Eurosurveillance* as supporting information alongside the article "Evidence for Antimicrobial Stewardship and Reduced Antimicrobial Resistance in the Mid-West of Ireland, 2012 - 2023: Findings from a One Health Study", on behalf of the authors, who remain responsible for the accuracy and appropriateness of the content. The same standards for ethics, copyright, attributions and permissions as for the article apply. Supplements are not edited by *Eurosurveillance* and the journal is not responsible for the maintenance of any links or email addresses provided therein.

		n	% Female
Hospital	Total	36038	79.0%
	Adult	31675	80.2%
	Paed	4363	70.4%
Community	Total	82516	88.5%
	Adult	74879	88.4%
	Paed	7637	89.3%
Other	Total	3865	82.1%
	Adult	3742	82.0%
	Paed	123	87.8%
	Total	122419	85.5%

Supplementary Table S1. Human urine isolate patient demographics, 2012 – 2023, Mid-West of Ireland. "Paed" (paediatric) patients were aged less than 15 years, adult patients were those aged 15 years and older.

		n	% Female
Hospital Acquired	Total	450	44.7%
	Adult	444	44.8%
	Paed	6	33.3%
Community Acquired	Total	1923	53.4%
	Adult	1875	53.5%
	Paed	48	47.9%
Total	Total	2373	51.7%
	Adult	2319	51.9%
	Paed	54	46.3%

Supplementary Table S2. Human blood isolate patient demographics, 2012 – 2023, Mid-West of Ireland. "Paed" (paediatric) patients were aged less than 15 years, adult patients were those aged 15 years and older.

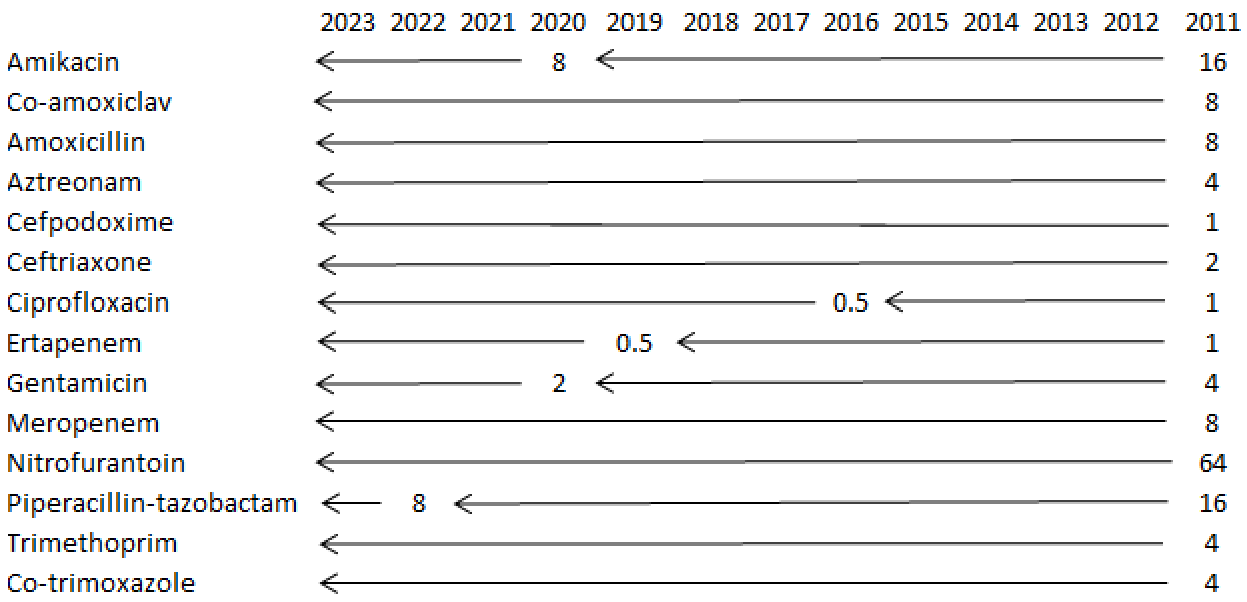

Supplementary Figure S1. Breakpoint changes made to EUCAST guidelines over the duration of the study period. Units in μg/ml

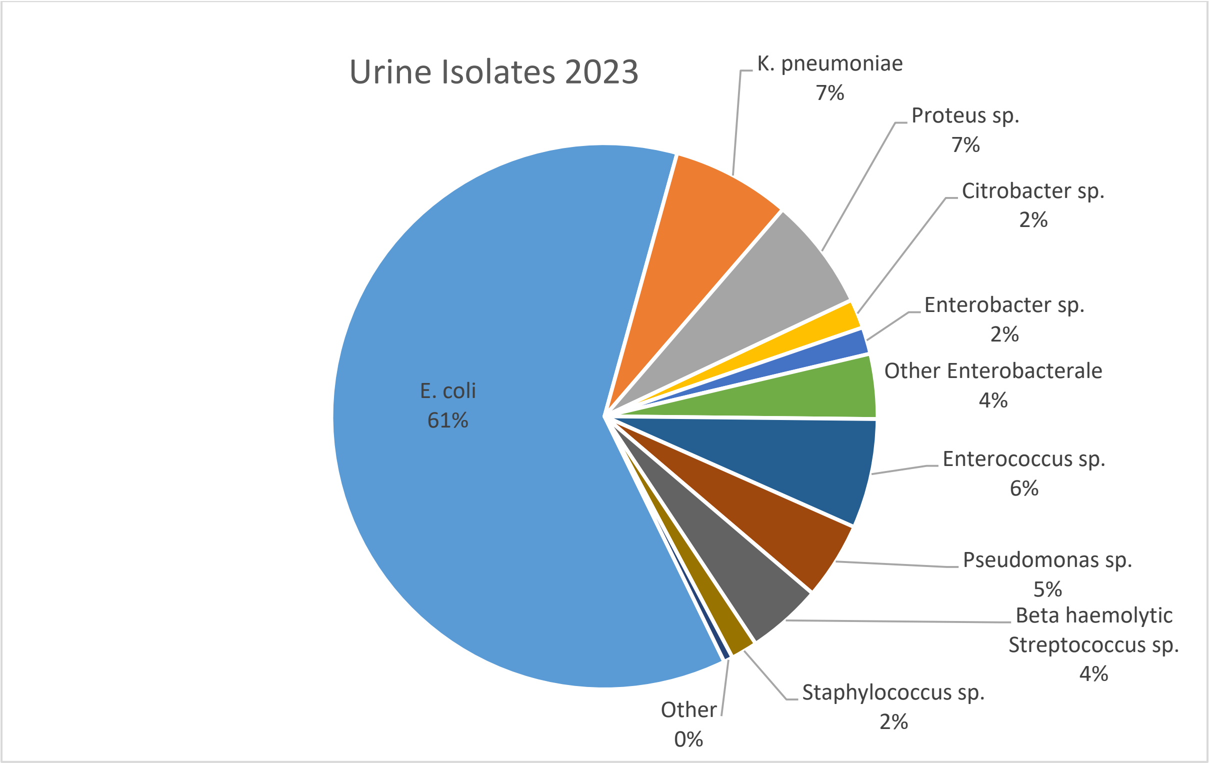

Supplementary Figure S2. The proportion of human urinary isolates detected from all urine samples in the Microbiology Laboratory at University Hospital Limerick (Ireland) in 2023, broken down by species.

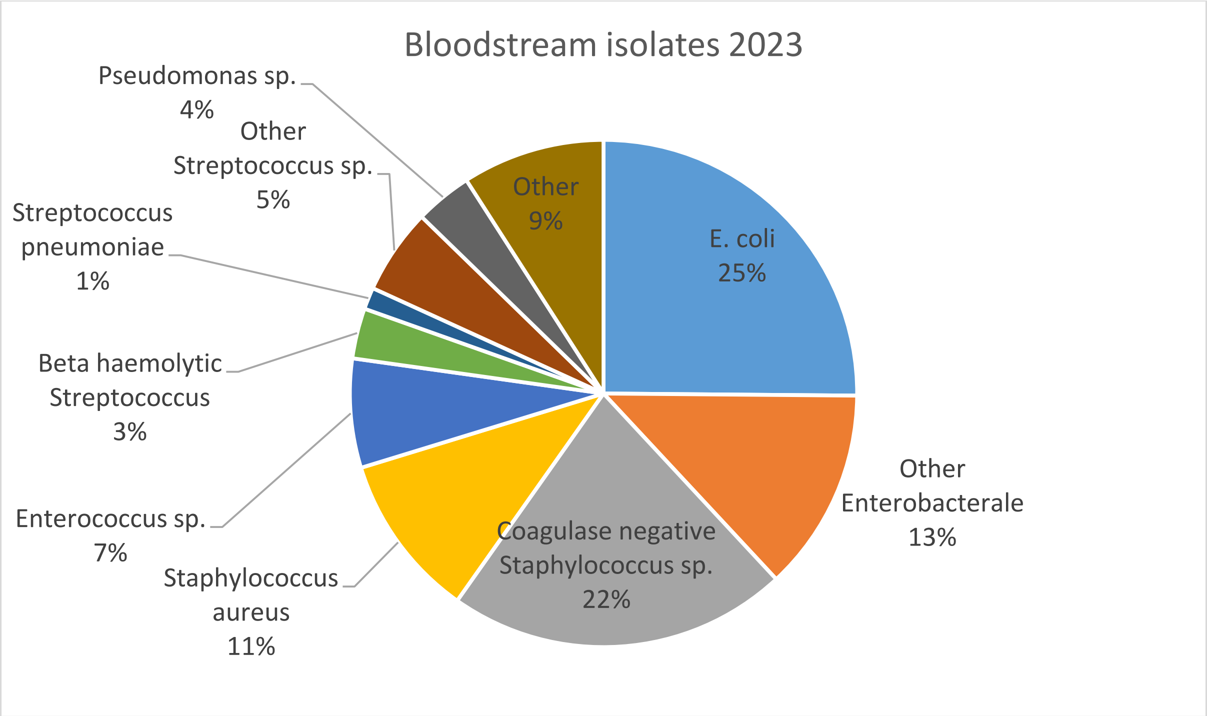

Supplementary Figure S3. The proportion of all human bloodstream isolates from the Microbiology Laboratory at University Hospital Limerick (Ireland) in 2023 broken down by species.

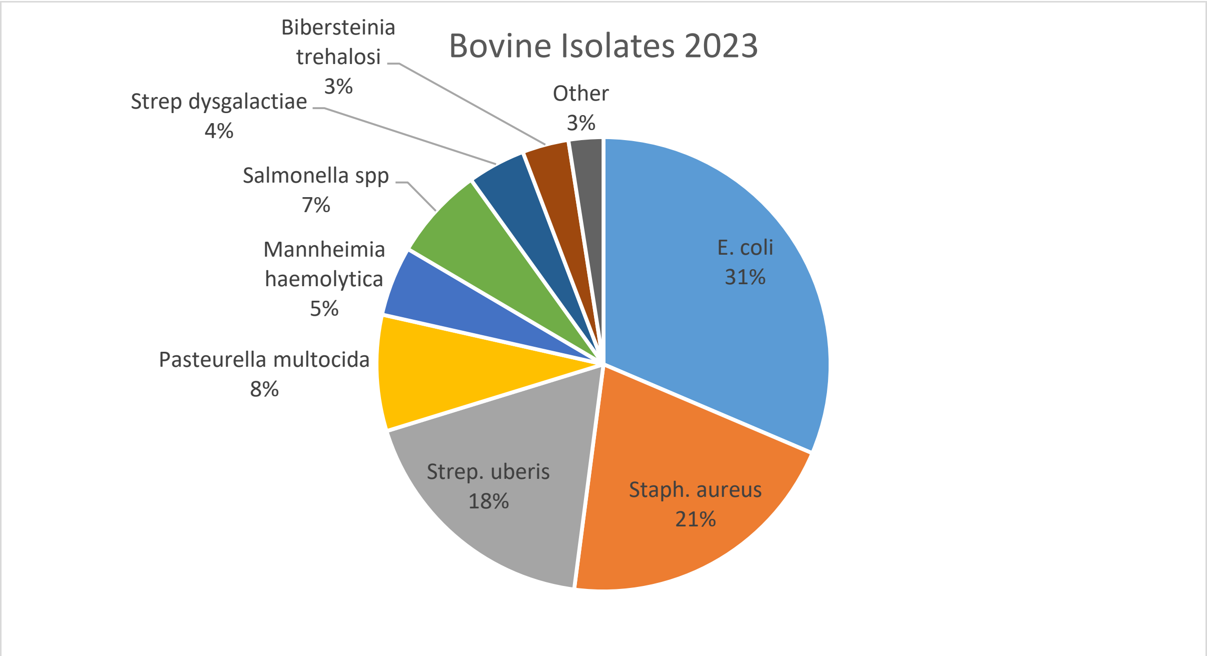

Supplementary Figure S4. The proportion of bovine isolates in the Regional Veterinary hospital Limerick (Ireland) in 2023 broken down by species.

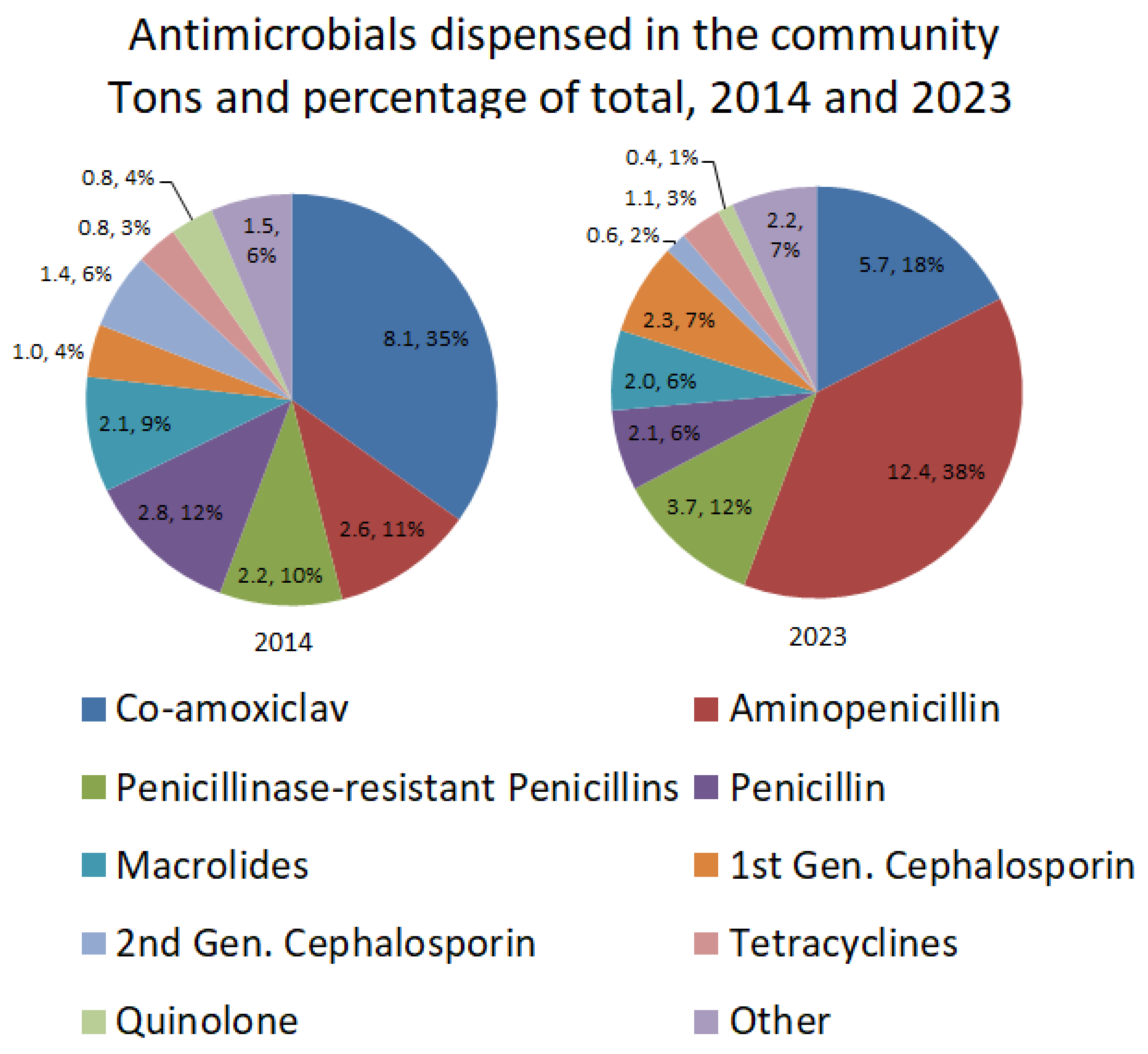

Supplementary Figure S5. Proportion of antimicrobials dispensed in community healthcare, Mid-West of Ireland, 2014 and 2023. Data supplied by the Primary Care Reimbursement Service (PCRS), with separate data for four reimbursement schemes which were pooled for this study: The Drug Payment Scheme, the General Medical Services Scheme, the High Tech Drug Scheme and the Long-Term Illness Scheme.

		2012	2013	2014	2015	2016	2017	2018	2019	2020	2021	2022	2023	Total	% Change	Prob > |z|	Z value
Amikacin	Resistant	0	1	3	3	0	0	1	0	0	0	0	0	8			
	Total	160	169	199	188	174	192	192	206	186	220	208	234	2328			
	% Resistant	0.00%	0.60%	1.50%	1.60%	0.00%	0.00%	0.50%	0.00%	0.00%	0.00%	0.00%	0.00%	0.30%	-1.10%	0.012	-2.503
Amoxicillin	Resistant	129	135	148	145	133	135	142	139	110	139	123	155	1633			
	Total	173	169	212	200	180	192	192	206	186	220	208	234	2372			
	% Resistant	74.60%	79.90%	69.80%	72.50%	73.90%	70.30%	74.00%	67.50%	59.10%	63.20%	59.10%	66.20%	68.80%	-16.50%	0.000	-5.006
Amoxicillin / Clavulanic acid	Resistant	59	103	117	114	97	106	100	102	80	97	76	102	1153			
	Total	173	169	212	200	180	192	192	206	186	220	208	234	2372			
	% Resistant	34.10%	60.90%	55.20%	57.00%	53.90%	55.20%	52.10%	49.50%	43.00%	44.10%	36.50%	43.60%	48.60%	-12.60%	0.000	-3.549
Aztreonam	Resistant	11	31	50	4	27	30	29	25	19	18	16	20	280			
	Total	155	169	211	23	135	192	192	206	186	220	208	234	2131			
	% Resistant	7.10%	18.30%	23.70%	17.40%	20.00%	15.60%	15.10%	12.10%	10.20%	8.20%	7.70%	8.50%	13.10%	-11.30%	0.000	-4.530
Ceftriaxone	Resistant	25	40	53	45	37	36	36	35	27	23	17	23	397			
	Total	158	169	199	187	173	191	191	206	186	220	208	233	2321			
	% Resistant	15.80%	23.70%	26.60%	24.10%	21.40%	18.80%	18.80%	17.00%	14.50%	10.50%	8.20%	9.90%	17.10%	-16.90%	0.000	-6.207
Ciprofloxacin	Resistant	58	62	78	70	57	65	57	52	43	41	33	45	661			
	Total	174	169	212	200	180	192	192	206	186	220	208	234	2373			
	% Resistant	33.30%	36.70%	36.80%	35.00%	31.70%	33.90%	29.70%	25.20%	23.10%	18.60%	15.90%	19.20%	27.90%	-23.60%	0.000	-7.400
Ertapenem	Resistant	0	0	0	1	0	0	1	0	1	2	0	0	5			
	Total	31	169	212	200	180	192	192	206	186	220	207	233	2228			
	% Resistant	0.00%	0.00%	0.00%	0.50%	0.00%	0.00%	0.50%	0.00%	0.50%	0.90%	0.00%	0.00%	0.20%	0.20%	0.530	0.627
Gentamicin	Resistant	25	35	50	47	35	32	24	35	19	29	16	25	372			
	Total	173	169	212	200	180	192	192	206	186	220	208	235	2373			
	% Resistant	14.50%	20.70%	23.60%	23.50%	19.40%	16.70%	12.50%	17.00%	10.20%	13.20%	7.70%	10.60%	15.70%	-13.40%	0.000	-5.195
Meropenem	Resistant	0	0	0	0	0	0	0	0	1	0	0	0	1			
	Total	174	169	205	193	180	192	192	206	186	220	208	235	2360			
	% Resistant	0.00%	0.00%	0.00%	0.00%	0.00%	0.00%	0.00%	0.00%	0.50%	0.00%	0.00%	0.00%	0.00%	0.10%	0.516	0.649
Piperacillin / Tazobactam	Resistant	36	44	23	23	15	18	23	19	10	24	26	26	287			
	Total	174	169	212	196	179	192	192	206	186	213	207	234	2360			
	% Resistant	20.70%	26.00%	10.80%	11.70%	8.40%	9.40%	12.00%	9.20%	5.40%	11.30%	12.60%	11.10%	12.20%	-9.30%	0.000	-3.990
Trimethoprim / Sulfamethoxazole	Resistant	71	68	76	77	66	61	66	71	49	67	57	78	807			
	Total	157	169	199	186	173	191	190	206	186	219	206	232	2314			
	% Resistant	45.20%	40.20%	38.20%	41.40%	38.20%	31.90%	34.70%	34.50%	26.30%	30.60%	27.70%	33.60%	34.90%	-15.10%	0.000	-4.374

Supplementary Table S3. Human bloodstream isolate (all) resistance rates, Mid-West of Ireland 2012 to 2023.

		2012	2013	2014	2015	2016	2017	2018	2019	2020	2021	2022	2023	Total	% change	Prob > |z|	Z value
Amikacin	Resistant	0	1	3	1	0	0	1	0	0	0	0	0	6			
	Total	39	34	49	33	35	39	38	35	33	47	33	27	442			
	% Resistant	0.00%	2.90%	6.10%	3.00%	0.00%	0.00%	2.60%	0.00%	0.00%	0.00%	0.00%	0.00%	1.40%	-3.7	0.054	-1.928
Amoxicillin	Resistant	36	28	40	27	28	29	29	26	24	31	21	17	336			
	Total	41	34	51	37	35	39	38	35	33	47	33	27	450			
	% Resistant	87.80%	82.40%	78.40%	73.00%	80.00%	74.40%	76.30%	74.30%	72.70%	66.00%	63.60%	63.00%	74.70%	-22.8	0.002	-3.151
Amoxicillin / Clavulanic acid	Resistant	16	24	34	23	21	24	21	23	15	26	15	12	254			
	Total	41	34	51	37	35	39	38	35	33	47	33	27	450			
	% Resistant	39.00%	70.60%	66.70%	62.20%	60.00%	61.50%	55.30%	65.70%	45.50%	55.30%	45.50%	44.40%	56.40%	-11.3	0.169	-1.375
Aztreonam	Resistant	2	8	14	1	6	5	6	6	3	2	1	2	56			
	Total	34	34	51	4	25	39	38	35	33	47	33	27	400			
	% Resistant	5.90%	23.50%	27.50%	25.00%	24.00%	12.80%	15.80%	17.10%	9.10%	4.30%	3.00%	7.40%	14.00%	-18.1	0.003	-3.008
Ceftriaxone	Resistant	6	10	20	10	11	7	7	8	4	3	2	2	90			
	Total	39	34	49	32	35	39	38	35	33	47	33	26	440			
	% Resistant	15.40%	29.40%	40.80%	31.30%	31.40%	17.90%	18.40%	22.90%	12.10%	6.40%	6.10%	7.70%	20.50%	-28.5	0.000	-4.194
Ciprofloxacin	Resistant	16	15	21	14	15	17	13	11	8	7	7	7	151			
	Total	41	34	51	37	35	39	38	35	33	47	33	27	450			
	% Resistant	39.00%	44.10%	41.20%	37.80%	42.90%	43.60%	34.20%	31.40%	24.20%	14.90%	21.20%	25.90%	33.60%	-28.1	0.000	-3.580
Ertapenem	Resistant	0	0	0	0	0	0	0	0	1	1	0	0	2			
	Total	4	34	51	37	35	39	38	35	33	47	33	26	412			
	% Resistant	0.00%	0.00%	0.00%	0.00%	0.00%	0.00%	0.00%	0.00%	3.00%	2.10%	0.00%	0.00%	0.50%	1.6	0.210	1.254
Gentamicin	Resistant	4	9	9	14	8	4	5	10	2	6	3	4	78			
	Total	41	34	51	37	35	39	38	35	33	47	33	27	450			
	% Resistant	9.80%	26.50%	17.60%	37.80%	22.90%	10.30%	13.20%	28.60%	6.10%	12.80%	9.10%	14.80%	17.30%	-10.5	0.095	-1.671
Meropenem	Resistant	0	0	0	0	0	0	0	0	1	0	0	0	1			
	Total	41	34	49	35	35	39	38	35	33	47	33	27	446			
	% Resistant	0.00%	0.00%	0.00%	0.00%	0.00%	0.00%	0.00%	0.00%	3.00%	0.00%	0.00%	0.00%	0.20%	0.6	0.426	0.797
Piperacillin / Tazobactam	Resistant	9	10	7	9	5	5	10	9	3	10	7	4	88			
	Total	41	34	51	37	35	39	38	35	33	44	33	27	447			
	% Resistant	22.00%	29.40%	13.70%	24.30%	14.30%	12.80%	26.30%	25.70%	9.10%	22.70%	21.20%	14.80%	19.70%	-3.3	0.623	-0.492
Trimethoprim / Sulfamethoxazole	Resistant	18	18	20	14	19	15	12	15	13	15	10	10	179			
	Total	38	34	49	32	35	39	38	35	33	47	33	26	439			
	% Resistant	47.40%	52.90%	40.80%	43.80%	54.30%	38.50%	31.60%	42.90%	39.40%	31.90%	30.30%	38.50%	40.80%	-18.3	0.028	-2.199

Supplementary Table S4. Human bloodstream isolate (hospital acquired source) resistance rates, Mid-West of Ireland 2012 to 2023.

		2012	2013	2014	2015	2016	2017	2018	2019	2020	2021	2022	2023	Total	% change	Prob > |z|	Z value
Amikacin	Resistant	0	0	0	2	0	0	0	0	0	0	0	0	2			
	Total	121	135	150	155	139	153	154	171	153	173	175	207	1886			
	% Resistant	0.00%	0.00%	0.00%	1.30%	0.00%	0.00%	0.00%	0.00%	0.00%	0.00%	0.00%	0.00%	0.10%	-0.3	0.233	-1.1920
Amoxicillin	Resistant	93	107	108	118	105	106	113	113	86	108	102	138	1297			
	Total	132	135	161	163	145	153	154	171	153	173	175	207	1922			
	% Resistant	70.50%	79.30%	67.10%	72.40%	72.40%	69.30%	73.40%	66.10%	56.20%	62.40%	58.30%	66.70%	67.50%	-14.4	0.000	-3.891
Amoxicillin / Clavulanic acid	Resistant	43	79	83	91	76	82	79	79	65	71	61	90	899			
	Total	132	135	161	163	145	153	154	171	153	173	175	207	1922			
	% Resistant	32.60%	58.50%	51.60%	55.80%	52.40%	53.60%	51.30%	46.20%	42.50%	41.00%	34.90%	43.50%	46.80%	-11.9	0.003	-3.029
Aztreonam	Resistant	9	23	36	3	21	25	23	19	16	16	15	18	224			
	Total	121	135	160	19	110	153	154	171	153	173	175	207	1731			
	% Resistant	7.40%	17.00%	22.50%	15.80%	19.10%	16.30%	14.90%	11.10%	10.50%	9.20%	8.60%	8.70%	12.90%	-9.8	0.000	-3.542
Ceftriaxone	Resistant	19	30	33	35	26	29	29	27	23	20	15	21	307			
	Total	119	135	150	155	138	152	153	171	153	173	175	207	1881			
	% Resistant	16.00%	22.20%	22.00%	22.60%	18.80%	19.10%	19.00%	15.80%	15.00%	11.60%	8.60%	10.10%	16.30%	-13.9	0.000	-4.683
Ciprofloxacin	Resistant	42	47	57	56	42	48	44	41	35	34	26	38	510			
	Total	133	135	161	163	145	153	154	171	153	173	175	207	1923			
	% Resistant	31.60%	34.80%	35.40%	34.40%	29.00%	31.40%	28.60%	24.00%	22.90%	19.70%	14.90%	18.40%	26.50%	-21.9	0.000	-6.301
Ertapenem	Resistant	0	0	0	1	0	0	1	0	0	1	0	0	3			
	Total	27	135	161	163	145	153	154	171	153	173	174	207	1816			
	% Resistant	0.00%	0.00%	0.00%	0.60%	0.00%	0.00%	0.60%	0.00%	0.00%	0.60%	0.00%	0.00%	0.20%	0.0	0.927	-0.092
Gentamicin	Resistant	21	26	41	33	27	28	19	25	17	23	13	21	294			
	Total	132	135	161	163	145	153	154	171	153	173	175	208	1923			
	% Resistant	15.90%	19.30%	25.50%	20.20%	18.60%	18.30%	12.30%	14.60%	11.10%	13.30%	7.40%	10.10%	15.30%	-13.9	0.000	-4.909
Meropenem	Resistant	0	0	0	0	0	0	0	0	0	0	0	0	0			
	Total	133	135	156	158	145	153	154	171	153	173	175	208	1914			
	% Resistant	0.00%	0.00%	0.00%	0.00%	0.00%	0.00%	0.00%	0.00%	0.00%	0.00%	0.00%	0.00%	0.00%	0.0	N/A	0.000
Piperacillin / Tazobactam	Resistant	27	34	16	14	10	13	13	10	7	14	19	22	199			
	Total	133	135	161	159	144	153	154	171	153	169	174	207	1913			
	% Resistant	20.30%	25.20%	9.90%	8.80%	6.90%	8.50%	8.40%	5.80%	4.60%	8.30%	10.90%	10.60%	10.40%	-9.6	0.000	-4.002
Trimethoprim / Sulfamethoxazole	Resistant	53	50	56	63	47	46	54	56	36	52	47	68	628			
	Total	119	135	150	154	138	152	152	171	153	172	173	206	1875			
	% Resistant	44.50%	37.00%	37.30%	40.90%	34.10%	30.30%	35.50%	32.70%	23.50%	30.20%	27.20%	33.00%	33.50%	-13.6	0.000	-3.598

Supplementary TableS5. Human bloodstream isolate (community source) resistance rates, Mid-West of Ireland 2012 to 2023.

		2012	2013	2014	2015	2016	2017	2018	2019	2020	2021	2022	2023	Total	% change	Prob > |z|	Z value
Amikacin	Resistant	4	11	17	17	16	15	17	12	22	27	43	49	250			
	Total	9486	9439	8678	10052	11339	10909	10747	10668	9199	8892	10267	11293	120969			
	% Resistant	0.00%	0.10%	0.20%	0.20%	0.10%	0.10%	0.20%	0.10%	0.20%	0.30%	0.40%	0.40%	0.20%	0.3%	0.000	7.6000
Amoxicillin	Resistant	5761	5783	5558	6234	6976	6479	6416	6238	5268	4943	5722	6473	71851			
	Total	9487	9440	9249	10444	11681	10908	10747	10671	9199	8952	10266	11295	122339			
	% Resistant	60.70%	61.30%	60.10%	59.70%	59.70%	59.40%	59.70%	58.50%	57.30%	55.20%	55.70%	57.30%	58.70%	-5.6%	0.000	-11.4070
Amoxicillin / Clavulanic acid	Resistant	1000	3666	2468	1846	1899	1769	1736	2000	1708	1509	1625	1866	23092			
	Total	9488	9441	9249	10444	11680	10908	10745	10671	9201	9011	10205	11288	122331			
	% Resistant	10.50%	38.80%	26.70%	17.70%	16.30%	16.20%	16.20%	18.70%	18.60%	16.70%	15.90%	16.50%	18.90%	-8.0%	0.000	-20.2320
Aztreonam	Resistant	N/A	N/A	N/A	N/A	688	801	803	732	644	507	554	625	5354			
	Total	N/A	N/A	N/A	N/A	8040	10902	10707	10672	9195	8891	10266	11294	79967			
	% Resistant	N/A	N/A	N/A	N/A	8.60%	7.30%	7.50%	6.90%	7.00%	5.70%	5.40%	5.50%	6.70%	-5.2%	0.000	-11.1900
Cefpodoxime	Resistant	108	1323	1339	1395	1615	1311	1324	1199	1055	952	1108	1257	13986			
	Total	1096	9439	9244	10440	11677	10906	10744	10672	9201	8892	10267	11295	113873			
	% Resistant	9.90%	14.00%	14.50%	13.40%	13.80%	12.00%	12.30%	11.20%	11.50%	10.70%	10.80%	11.10%	12.30%	-4.3%	0.000	-11.6260
Cephalexin	Resistant	1391	1306	1349	1388	1570	1295	1327	1231	1065	953	1096	1238	15209			
	Total	9455	9440	9248	10443	11678	10907	10746	10671	9201	9012	10266	11293	122360			
	% Resistant	14.70%	13.80%	14.60%	13.30%	13.40%	11.90%	12.30%	11.50%	11.60%	10.60%	10.70%	11.00%	12.40%	-4.6%	0.000	-13.9610
Ciprofloxacin	Resistant	1869	2085	2146	2369	2595	2245	2243	1877	1493	1381	1422	1567	23292			
	Total	9489	9440	9250	10446	11684	10909	10747	10672	9200	9009	10263	11293	122402			
	% Resistant	19.70%	22.10%	23.20%	22.70%	22.20%	20.60%	20.90%	17.60%	16.20%	15.30%	13.90%	13.90%	19.00%	-10.2%	0.000	-25.8380
Ertapenem	Resistant	1	6	12	9	8	5	2	6	9	11	0	0	69			
	Total	1239	9439	9247	10442	11680	10907	10745	10673	9200	8864	10187	11206	113829			
	% Resistant	0.10%	0.10%	0.10%	0.10%	0.10%	0.00%	0.00%	0.10%	0.10%	0.10%	0.00%	0.00%	0.10%	-0.1%	0.006	-2.7320
Gentamicin	Resistant	794	1063	1093	1215	1223	994	1040	955	858	826	781	930	11772			
	Total	9489	9441	9250	10446	11684	10910	10746	10672	9201	9013	10267	11295	122414			
	% Resistant	8.40%	11.30%	11.80%	11.60%	10.50%	9.10%	9.70%	8.90%	9.30%	9.20%	7.60%	8.20%	9.60%	-2.9%	0.000	-9.9480
Meropenem	Resistant	0	0	2	0	0	0	0	0	0	0	1	0	3			
	Total	9488	9441	8702	10062	11683	10908	10747	10673	9201	9013	10267	11295	121480			
	% Resistant	0.00%	0.00%	0.00%	0.00%	0.00%	0.00%	0.00%	0.00%	0.00%	0.00%	0.00%	0.00%	0.00%	0.0%	0.640	-0.4680
Nitrofurantoin	Resistant	198	202	203	273	181	152	175	160	196	201	198	221	2360			
	Total	9489	9441	9248	10444	11680	10906	10746	10672	9201	9013	10267	11295	122402			
	% Resistant	2.10%	2.10%	2.20%	2.60%	1.50%	1.40%	1.60%	1.50%	2.10%	2.20%	1.90%	2.00%	1.90%	-0.2%	0.103	-1.6280
Piperacillin / Tazobactam	Resistant	120	293	381	231	363	569	534	613	497	625	621	814	5661			
	Total	9486	9439	8804	1587	8328	10900	10745	10670	9196	9007	10267	11295	109724			
	% Resistant	1.30%	3.10%	4.30%	14.60%	4.40%	5.20%	5.00%	5.70%	5.40%	6.90%	6.00%	7.20%	5.20%	4.7%	0.000	20.5060
Trimethoprim	Resistant	3406	3453	3368	3712	4038	3751	3685	3649	3076	2850	3268	3750	42006			
	Total	9487	9440	9247	10443	11679	10906	10745	10670	9200	9012	10266	11292	122387			
	% Resistant	35.90%	36.60%	36.40%	35.50%	34.60%	34.40%	34.30%	34.20%	33.40%	31.60%	31.80%	33.20%	34.30%	-4.8%	0.000	-10.1250

Supplementary TableS6. Human urine isolate (all) resistance rates, Mid-West of Ireland 2012 to 2023.

		2012	2013	2014	2015	2016	2017	2018	2019	2020	2021	2022	2023	Total	% change	Prob > |z|	Z value
Amikacin	Resistant	2	7	8	7	10	3	11	4	8	10	10	25	105	0.4	0.000	4.2480
	Total	3095	3043	2643	3064	3239	3262	3008	2915	2473	2655	2974	3156	35527			
	% Resistant	0.10%	0.20%	0.30%	0.20%	0.30%	0.10%	0.40%	0.10%	0.30%	0.40%	0.30%	0.80%	0.30%			
Amoxicillin	Resistant	2015	1976	1801	2005	2184	2044	1938	1842	1510	1617	1773	1950	22655	-5.1	0.000	-5.7820
	Total	3094	3044	2821	3183	3372	3261	3008	2915	2472	2696	2974	3156	35996			
	% Resistant	65.10%	64.90%	63.80%	63.00%	64.80%	62.70%	64.40%	63.20%	61.10%	60.00%	59.60%	61.80%	62.90%			
Amoxicillin / Clavulanic acid	Resistant	370	1295	871	659	695	625	591	647	546	574	559	622	8054	-7.5	0.000	-9.7720
	Total	3095	3044	2821	3183	3371	3261	3008	2916	2473	2726	2959	3151	36008			
	% Resistant	12.00%	42.50%	30.90%	20.70%	20.60%	19.20%	19.60%	22.20%	22.10%	21.10%	18.90%	19.70%	22.40%			
Aztreonam	Resistant	N/A	N/A	N/A	N/A	246	264	271	259	203	183	200	217	1843	-5.5	0.000	-5.9090
	Total	N/A	N/A	N/A	N/A	2326	3259	2995	2916	2470	2654	2973	3156	22749			
	% Resistant	N/A	N/A	N/A	N/A	10.60%	8.10%	9.00%	8.90%	8.20%	6.90%	6.70%	6.90%	8.10%			
Cefpodoxime	Resistant	44	485	469	529	598	430	443	414	317	354	393	406	4882	-4.8	0.000	-7.9900
	Total	362	3044	2818	3182	3371	3261	3005	2916	2473	2655	2974	3156	33217			
	% Resistant	12.20%	15.90%	16.60%	16.60%	17.70%	13.20%	14.70%	14.20%	12.80%	13.30%	13.20%	12.90%	14.70%			
Cephalexin	Resistant	548	478	477	522	573	425	453	424	324	354	389	411	5378	-5.2	0.000	-0.0043
	Total	3082	3044	2820	3183	3369	3261	3007	2916	2473	2725	2973	3155	36008			
	% Resistant	17.80%	15.70%	16.90%	16.40%	17.00%	13.00%	15.10%	14.50%	13.10%	13.00%	13.10%	13.00%	14.90%			
Ciprofloxacin	Resistant	680	716	718	816	888	739	746	598	436	483	491	543	7854	-9.3	0.000	-12.3120
	Total	3095	3044	2822	3185	3374	3263	3008	2916	2473	2724	2973	3155	36032			
	% Resistant	22.00%	23.50%	25.40%	25.60%	26.30%	22.60%	24.80%	20.50%	17.60%	17.70%	16.50%	17.20%	21.80%			
Ertapenem	Resistant	0	2	0	1	2	2	2	0	2	2	0	0	13	0.0	0.550	-0.5970
	Total	417	3043	2820	3183	3371	3261	3006	2917	2473	2647	2947	3126	33211			
	% Resistant	0.00%	0.10%	0.00%	0.00%	0.10%	0.10%	0.10%	0.00%	0.10%	0.10%	0.00%	0.00%	0.00%			
Gentamicin	Resistant	292	349	375	420	407	369	359	320	251	286	230	294	3952	-3.0	0.000	-5.2450
	Total	3095	3044	2823	3185	3374	3263	3008	2916	2473	2726	2974	3156	36037			
	% Resistant	9.40%	11.50%	13.30%	13.20%	12.10%	11.30%	11.90%	11.00%	10.10%	10.50%	7.70%	9.30%	11.00%			
Meropenem	Resistant	0	0	1	0	0	0	0	0	0	0	1	0	2	0.003	0.820	0.2280
	Total	3095	3044	2655	3067	3374	3262	3008	2917	2473	2726	2974	3156	35751			
	% Resistant	0.00%	0.00%	0.00%	0.00%	0.00%	0.00%	0.00%	0.00%	0.00%	0.00%	0.00%	0.00%	0.00%			
Nitrofurantoin	Resistant	62	45	61	82	48	38	63	38	47	43	54	55	636	-0.28	0.247	-1.1590
	Total	3095	3043	2820	3183	3371	3260	3007	2916	2473	2726	2974	3156	36024			
	% Resistant	2.00%	1.50%	2.20%	2.60%	1.40%	1.20%	2.10%	1.30%	1.90%	1.60%	1.80%	1.70%	1.80%			
Piperacillin / Tazobactam	Resistant	53	106	147	190	184	204	214	215	175	248	251	301	2288	6.3	0.000	13.3880
	Total	3094	3043	2732	1334	2728	3260	3007	2916	2473	2722	2974	3156	33439			
	% Resistant	1.70%	3.50%	5.40%	14.20%	6.70%	6.30%	7.10%	7.40%	7.10%	9.10%	8.40%	9.50%	6.80%			
Trimethoprim	Resistant	1138	1138	1073	1125	1246	1130	1063	1030	812	880	948	1103	12686	-5.2	0.000	-5.8970
	Total	3094	3044	2819	3183	3370	3260	3006	2915	2472	2726	2974	3156	36019			
	% Resistant	36.80%	37.40%	38.10%	35.30%	37.00%	34.70%	35.40%	35.30%	32.80%	32.30%	31.90%	34.90%	35.20%			

Supplementary TableS7. Human urine isolate (acute hospital locations) resistance rates, Mid-West of Ireland 2012 to 2023.

		2012	2013	2014	2015	2016	2017	2018	2019	2020	2021	2022	2023	Total	% change	Prob > |z|	Z value
Amikacin	Resistant	2	3	9	8	5	12	6	7	14	16	31	22	135			
	Total	6038	6099	5728	6650	7743	7316	7447	7476	6457	5955	6972	7741	81622			
	% Resistant	0.00%	0.00%	0.20%	0.10%	0.10%	0.20%	0.10%	0.10%	0.20%	0.30%	0.40%	0.30%	0.20%	0.3%	0.000	6.4280
Amoxicillin	Resistant	3530	3625	3551	4003	4571	4234	4280	4228	3613	3166	3761	4269	46831			
	Total	6040	6099	6107	6913	7940	7316	7447	7479	6457	5970	6971	7743	82482			
	% Resistant	58.40%	59.40%	58.10%	57.90%	57.60%	57.90%	57.50%	56.50%	56.00%	53.00%	54.00%	55.10%	56.80%	-5.6%	0.000	-9.1300
Amoxicillin / Clavulanic acid	Resistant	601	2245	1487	1107	1143	1079	1088	1298	1100	871	999	1160	14178			
	Total	6040	6100	6107	6913	7940	7316	7446	7478	6458	5995	6925	7741	82459			
	% Resistant	10.00%	36.80%	24.30%	16.00%	14.40%	14.70%	14.60%	17.40%	17.00%	14.50%	14.40%	15.00%	17.20%	-8.0%	0.000	-17.2580
Aztreonam	Resistant	N/A	N/A	N/A	N/A	410	495	497	444	416	299	328	378	3267			
	Total	N/A	N/A	N/A	N/A	5456	7312	7421	7479	6456	5955	6972	7742	54793			
	% Resistant	N/A	N/A	N/A	N/A	7.50%	6.80%	6.70%	5.90%	6.40%	5.00%	4.70%	4.90%	6.00%	-4.8%	0.000	-8.9410
Cefpodoxime	Resistant	63	783	800	791	954	820	832	753	698	558	676	792	8520			
	Total	704	6098	6105	6910	7937	7315	7447	7479	6458	5955	6972	7743	77123			
	% Resistant	8.90%	12.80%	13.10%	11.40%	12.00%	11.20%	11.20%	10.10%	10.80%	9.40%	9.70%	10.20%	11.00%	-3.6%	0.000	-8.3940
Cephalexin	Resistant	774	778	799	792	940	808	822	773	704	555	670	770	9185			
	Total	6020	6099	6107	6912	7941	7316	7447	7478	6458	5997	6972	7742	82489			
	% Resistant	12.90%	12.80%	13.10%	11.50%	11.80%	11.00%	11.00%	10.30%	10.90%	9.30%	9.60%	9.90%	11.10%	-3.9%	0.000	-10.0420
Ciprofloxacin	Resistant	1086	1278	1310	1434	1592	1410	1411	1213	1000	839	881	962	14416			
	Total	6041	6099	6107	6913	7941	7315	7447	7479	6457	5995	6969	7742	82505			
	% Resistant	18.00%	21.00%	21.50%	20.70%	20.00%	19.30%	18.90%	16.20%	15.50%	14.00%	12.60%	12.40%	17.50%	-9.7%	0.000	-20.8320
Ertapenem	Resistant	1	4	11	7	5	3	0	5	7	9	0	0	52			
	Total	786	6099	6106	6911	7940	7315	7447	7479	6458	5936	6924	7688	77089			
	% Resistant	0.10%	0.10%	0.20%	0.10%	0.10%	0.00%	0.00%	0.10%	0.10%	0.20%	0.00%	0.00%	0.10%	-0.1%	0.010	-2.5700
Gentamicin	Resistant	454	670	667	737	767	590	636	605	579	496	517	598	7316			
	Total	6041	6100	6106	6913	7941	7316	7446	7479	6458	5997	6972	7743	82512			
	% Resistant	7.50%	11.00%	10.90%	10.70%	9.70%	8.10%	8.50%	8.10%	9.00%	8.30%	7.40%	7.70%	8.90%	-2.6%	0.000	-7.5690
Meropenem	Resistant	0	0	0	0	0	0	0	0	0	0	0	0	0			
	Total	6040	6100	5737	6656	7940	7315	7447	7479	6458	5997	6972	7743	81884			
	% Resistant	0.00%	0.00%	0.00%	0.00%	0.00%	0.00%	0.00%	0.00%	0.00%	0.00%	0.00%	0.00%	0.00%	0.0%	N/A	0.0000
Nitrofurantoin	Resistant	125	153	126	179	130	108	106	116	144	154	138	157	1636			
	Total	6041	6101	6107	6913	7940	7316	7447	7479	6458	5997	6972	7743	82514			
	% Resistant	2.10%	2.50%	2.10%	2.60%	1.60%	1.50%	1.40%	1.60%	2.20%	2.60%	2.00%	2.00%	2.00%	-0.2%	0.363	-0.9100
Piperacillin / Tazobactam	Resistant	59	174	207	16	165	343	302	378	298	348	345	481	3116			
	Total	6040	6099	5758	154	5313	7309	7446	7477	6454	5995	6972	7743	72760			
	% Resistant	1.00%	2.90%	3.60%	10.40%	3.10%	4.70%	4.10%	5.10%	4.60%	5.80%	4.90%	6.20%	4.30%	4.3%	0.000	16.6330
Trimethoprim	Resistant	2112	2193	2171	2449	2644	2493	2490	2523	2172	1881	2213	2498	27839			
	Total	6040	6099	6107	6912	7941	7316	7447	7478	6458	5996	6971	7740	82505			
	% Resistant	35.00%	36.00%	35.50%	35.40%	33.30%	34.10%	33.40%	33.70%	33.60%	31.40%	31.70%	32.30%	33.70%	-4.3%	0.000	-7.4420

Supplementary Table S8. Human urine isolate (community/general practice locations) resistance rates, Mid-West of Ireland 2012 to 2023.

		2012	2013	2014	2015	2016	2017	2018	2019	2020	2021	2022	2023	Total	% change	Prob > |z|	Z value
Amoxycillin / Clavulanic Acid	Resistant	31	16	10	12	9	18	4	3	8	3	3	10	127			
	Total	74	55	58	51	47	73	41	46	30	25	45	38	583			
	% Resistant	41.90%	29.10%	17.20%	23.50%	19.10%	24.70%	9.80%	6.50%	26.70%	12.00%	6.70%	26.30%	21.80%	-22.9	0.001	-3.837
Ampicillin	Resistant	46	27	25	16	23	30	10	16	16	8	18	25	260			
	Total	74	55	58	51	47	73	41	46	30	25	47	38	585			
	% Resistant	62.20%	49.10%	43.10%	31.40%	48.90%	41.10%	24.40%	34.80%	53.30%	32.00%	38.30%	65.80%	44.40%	-8.6	0.230	-1.199
Cefpodoxime	Resistant	0	2	2	3	1	6	0	0	0	0	1	1	16			
	Total	71	54	58	51	46	71	40	45	30	24	45	38	573			
	% Resistant	0.00%	3.70%	3.40%	5.90%	2.20%	8.50%	0.00%	0.00%	0.00%	0.00%	2.20%	2.60%	2.80%	-1.2	0.629	-0.483
Ceftiofur	Resistant	2	0	0	0	2	4	0	0	0	0	1	1	10			
	Total	74	55	58	51	47	73	41	46	30	24	45	38	582			
	% Resistant	2.70%	0.00%	0.00%	0.00%	4.30%	5.50%	0.00%	0.00%	0.00%	0.00%	2.20%	2.60%	1.70%	0.2	0.895	0.132
Cephalothin	Resistant	7	7	4	8	4	9	5	6	3	0	3	2	58			
	Total	40	38	43	41	29	52	33	40	18	16	34	15	399			
	% Resistant	17.50%	18.40%	9.30%	19.50%	13.80%	17.30%	15.20%	15.00%	16.70%	0.00%	8.80%	13.30%	14.50%	-7.9	0.230	-1.201
Enrofloxacin	Resistant	9	0	4	5	10	15	5	3	10	4	2	8	75			
	Total	73	55	58	51	46	71	40	45	30	25	47	38	579			
	% Resistant	12.30%	0.00%	6.90%	9.80%	21.70%	21.10%	12.50%	6.70%	33.30%	16.00%	4.30%	21.10%	13.00%	9.4	0.052	1.941
Kanamycin	Resistant	10	5	6	6	6	5	3	3	1	3	3	3	54			
	Total	40	39	43	41	29	52	33	40	18	16	35	15	401			
	% Resistant	25.00%	12.80%	14.00%	14.60%	20.70%	9.60%	9.10%	7.50%	5.60%	18.80%	8.60%	20.00%	13.50%	-10.1	0.108	-1.605
Neomycin	Resistant	26	13	12	11	11	16	5	5	3	4	5	10	121			
	Total	72	54	57	51	46	71	40	45	30	25	47	38	576			
	% Resistant	36.10%	24.10%	21.10%	21.60%	23.90%	22.50%	12.50%	11.10%	10.00%	16.00%	10.60%	26.30%	21.00%	-18.0	0.002	-3.042
Streptomycin	Resistant	40	22	20	18	23	24	9	9	13	7	16	23	224			
	Total	72	54	56	51	46	71	40	45	30	24	45	38	572			
	% Resistant	55.60%	40.70%	35.70%	35.30%	50.00%	33.80%	22.50%	20.00%	43.30%	29.20%	35.60%	60.50%	39.20%	-8.3	0.243	-1.167
Sulphamethoxazole / Trimethoprim	Resistant	21	10	11	7	14	20	7	9	14	5	8	15	141			
	Total	74	55	58	51	47	73	41	46	30	25	47	38	585			
	% Resistant	28.40%	18.20%	19.00%	13.70%	29.80%	27.40%	17.10%	19.60%	46.70%	20.00%	17.00%	39.50%	24.10%	7.2	0.242	1.170
Tetracycline	Resistant	42	24	21	18	24	25	10	8	12	11	14	23	232			
	Total	74	55	58	51	47	73	41	46	30	25	45	38	583			
	% Resistant	56.80%	43.60%	36.20%	35.30%	51.10%	34.20%	24.40%	17.40%	40.00%	44.00%	31.10%	60.50%	39.80%	-10.6	0.136	-1.492

*Supplementary Table S9. Bovine isolate resistance rates.*No EUCAST breakpoints available, CLSI criteria used for interpretation.¥Omitted from analysis because no comparable data were available from human isolates.*

	3GC	3GC	FQ	FQ	Carb	Carb	Gent	Gent	AZT	AZT	OAUG	OAUG	IVAUG	IVAUG	PIT	PIT
	Hosp	National Median	Hosp	National Median	Hosp	National Median	Hosp	National Median	Hosp	National Median	Hosp	National Median	Hosp	National Median	Hosp	National Median
2007	1.89	1.19	8.64	8.54	0.45	0.22	2.23	1.32	0.07	0.00	6.51	7.64	6.07	5.51	3.05	2.63
2008	1.48	1.19	7.45	6.89	0.68	0.34	2.17	1.41	0.04	0.00	6.78	7.81	5.94	5.42	3.73	3.12
2009	1.31	1.33	6.01	5.25	0.82	0.51	2.23	1.45	0.05	0.00	6.31	7.95	6.96	5.28	4.10	3.82
2010	1.74	1.22	4.54	4.77	1.32	0.62	1.98	1.62	0.06	0.00	8.52	8.66	8.58	5.59	5.07	4.04
2011	1.52	1.27	5.19	4.85	1.42	0.67	2.29	1.75	0.07	0.00	9.20	8.87	8.69	5.96	5.45	4.99
2012	1.46	1.37	5.64	5.26	2.08	1.02	2.39	1.93	0.12	0.00	10.52	8.97	9.12	6.39	5.96	5.22
2013	1.49	1.19	6.22	4.56	2.58	1.32	2.80	1.90	0.04	0.01	9.87	9.30	8.75	6.16	6.79	6.10
2014	1.48	1.42	5.74	4.71	2.65	1.24	2.91	2.04	0.15	0.02	9.05	8.61	7.82	5.66	7.76	6.19
2015	1.35	1.42	5.68	4.25	1.87	1.32	2.71	2.07	0.10	0.03	8.87	8.31	7.81	5.92	7.82	7.35
2016	1.69	1.49	5.04	4.54	1.49	1.44	2.57	1.98	0.25	0.06	9.55	8.26	7.60	5.97	8.90	8.27
2017	1.93	2.14	5.05	4.49	2.20	1.18	2.92	2.51	0.24	0.04	9.25	8.61	9.27	7.11	8.29	6.77
2018	2.02	2.22	5.42	4.42	2.34	0.95	2.47	2.27	0.16	0.07	8.91	8.42	9.68	6.97	10.33	7.70
2019	2.37	2.28	4.18	2.93	2.28	0.96	2.58	2.52	0.23	0.04	8.62	8.36	9.18	6.60	10.88	7.32
2020	2.12	2.18	3.90	2.35	1.73	1.19	2.09	2.18	0.23	0.12	7.62	7.09	9.49	6.08	10.81	7.54
2021	2.28	2.53	2.64	2.11	2.40	1.48	1.87	2.06	0.25	0.12	7.58	6.40	9.65	5.72	11.32	8.49
2022	2.47	2.66	2.45	2.03	2.50	1.50	1.71	2.09	0.54	0.17	8.37	7.82	8.89	6.39	12.66	8.72

Supplementary Table S6. Human Hospital antimicrobial consumption report, expressed as defined daily dose per 100 in-patient bed days used. “Hosp” is the combined result for UL Hospitals group, “National median” is the median rate among all hospitals in Ireland. “3GC” are third generation cephalosporins, “FQ” are fluoroquinolones, “Carb” are carbapenems, “gent is the aminoglycoside gentamicin, “AZT” is the monobactam aztreonam, “OAUG” and “IVAUG” are the oral and intravenous formulations of the aminopenicillin/beta lactam inhibitor co-amoxiclav, “PIT” is the extended-spectrum penicillin/beta-lactamase inhibitor piperacillin/tazobactam. Adapted from HPSC MicroB project data to Q1/Q2 2023, Health Protection Surveillance Centre, www.hpsc.ie.
